# Characterization of Poly(Acrylic) Acid-Modified Heterogenous Anion Exchange Membranes with Improved Monovalent Permselectivity for RED

**DOI:** 10.3390/membranes10060134

**Published:** 2020-06-26

**Authors:** Ivan Merino-Garcia, Francis Kotoka, Carla A.M. Portugal, João G. Crespo, Svetlozar Velizarov

**Affiliations:** Associated Laboratory for Green Chemistry—Clean Technologies and Processes (LAQV), REQUIMTE, Chemistry Department, FCT, Universidade Nova de Lisboa, 2829-516 Caparica, Portugal; ime.garcia@fct.unl.pt (I.M.-G.); franciskotoka90@gmail.com (F.K.); cmp@fct.unl.pt (C.A.M.P.); jgc@fct.unl.pt (J.G.C.)

**Keywords:** anion exchange membranes, poly(acrylic) acid modification, monovalent permselective membranes, antifouling strategies, reverse electrodialysis

## Abstract

The performance of anion-exchange membranes (AEMs) in Reverse Electrodialysis is hampered by both presence of multivalent ions and fouling phenomena, thus leading to reduced net power density. Therefore, we propose a monolayer surface modification procedure to functionalize Ralex-AEMs with poly(acrylic) acid (PAA) in order to (i) render a monovalent permselectivity, and (ii) minimize organic fouling. Membrane surface modification was carried out by putting heterogeneous AEMs in contact with a PAA-based aqueous solution for 24 h. The resulting modified membranes were firstly characterized by contact angle, water uptake, ion exchange capacity, fixed charge density, and swelling degree measurements, whereas their electrochemical responses were evaluated through cyclic voltammetry. Besides, their membrane electro-resistance was also studied via electrochemical impedance spectroscopy analyses. Finally, membrane permselectivity and fouling behavior in the presence of humic acid were evaluated through mass transport experiments using model NaCl containing solutions. The use of modified PAA-AEMs resulted in a significantly enhanced monovalent permselectivity (sulfate rejection improved by >35%) and membrane hydrophilicity (contact angle decreased by >15%) in comparison with the behavior of unmodified Ralex-AEMs, without compromising the membrane electro-resistance after modification, thus demonstrating the technical feasibility of the proposed membrane modification procedure. This study may therefore provide a feasible way for achieving an improved Reverse Electrodialysis process efficiency.

## 1. Introduction

The continuous rise of worldwide electricity demand has led to an increasing global interest in the study and development of green technologies capable of generating sustainable and renewable power [1,2]. In this respect, Reverse Electrodialysis (RED) represents an attractive technology due to the possibility of harvesting renewable energy from salinity gradients (e.g., between seawater and river water) through the use of alternating anion exchange membranes (AEMs) and cation exchange membranes (CEMs) forming cell pairs, where the different compartments between these membranes are fed with streams of different salinity (feedwaters with high and low salt concentration) [3,4,5]. Salinity gradient is, therefore, the driving force for the transport of ions from one compartment to the adjacent ones, thus creating an ionic current, which can be converted into electrical current by using electrodes, at which reversible redox reactions occur [6]. However, the practical application of this technology is currently limited by the presence of divalent ions in natural streams [2,4,7,8], which decreases the obtainable net power density according to Nernst equation as follows:

(1) $$OCV=\frac{NRT}{F}\left[\frac{\alpha_{CEM}}{z_{CEM}}\ln\frac{\gamma_{c,CEM}\;c_{c}}{\gamma_{d,CEM}\;c_{d}}+\frac{\alpha_{AEM}}{z_{AEM}}\ln\frac{\gamma_{c,AEM}\;c_{c}}{\gamma_{d,AEM}\;c_{d}}\right]$$

where *OCV* represents the open circuit voltage, *N* is the number of cell pairs, *R* the universal gas constant, *T* the absolute temperature, *F* the Faraday constant, α is equal to the permselectivity of the corresponding ion exchange membrane, *γ* represents the activity coefficient (where subscripts *c* and *d* stand for concentrate and dilute saline solutions, respectively), *c* is the molar concentration, and *z* is equal to the valence of the anion/cation that crosses the corresponding membrane. Accordingly, the higher the *z*, the lower the *OCV*, thus leading to reduced obtainable power output. As a result, the development of mono-selective AEMs and CEMs is crucial for an improved RED process efficiency. Besides, new efforts focusing on optimizing the operation variables of RED systems and their effects on the overall internal resistance, gross power and *OCV*, among others, have been recently considered to move forward into the large-scale implementation of this technology [9,10]. 

Moreover, different fouling-based phenomena such as organic fouling and scaling that negatively affects ion exchange membranes performance, promotes a significant loss (with time) of the generated power density [11]. Therefore, fouling control represents one of the main challenges to be addressed for a successful industrial implementation of the RED technology [6,12,13,14]. Although fouling issues can be investigated at stack level [15], most research is mainly focused on membrane level [13,16,17], especially regarding AEMs due to the negative charge of natural organic matter (NOM) such as humic acids, creating undesirable interactions between the fixed positively charged groups of AEMs and such foulant materials that hamper the performance of the process. In this respect, the presence of NOM has been demonstrated to present a larger impact on the obtainable net power density than the ionic composition [18].

In this context, surface modification of AEMs represents one of the most promising strategies to render a monovalent permselectivity as well as increasing fouling resistance [19,20,21]. Although different modification techniques such as polymerization by UV-irradiation [22] or chemical oxidation [23], among others, have been addressed in literature in an attempt to overcome the so-mentioned limitations, the possibility of incorporating a negative hydrophilic layer (or multilayers) on an AEM surface represents an attractive option for the electro-membrane processes field [24], because such a layer would act not only as a multivalent ions rejection wall, but also favoring monovalent ions passage owing to the Donnan effect [17,25], and preventing fouling because of its hydrophilic properties and negative charge at the same time [26]. As recently reviewed [1], direct casting, dip coating, immersion, and layer-by-layer (LbL) deposition, among others, are the most common available approaches considered to incorporate a beneficial hydrophilic layer on a membrane surface. In addition, due to the importance of selecting an appropriate surface modifying agent, a wide variety of interesting and feasible alternatives, ranging from polymers and biopolymers to nanoparticles and ionic liquids, have been already proposed. For example, focusing on the development of polymers and biopolymers, on the one hand, both poly(sodium 4-styrene sulfonate) (PSS) and poly(ethylenimine) (PEI)-based aqueous solutions were used as AEM modifying agents via the LbL method, demonstrating an improvement of mono-selectivity and antifouling properties, highlighting the importance of controlling both modifying agent concentration and deposition time, with the purpose of producing thinner membranes with lower resistance for an improved RED power performance [2]. On the other hand, the application of biopolymers such as chitosan-based materials are emerging for functionalizing AEMs, owing to their easiness to create thin layers on membrane surface as well as their multiple beneficial associated properties such as biodegradability, stability, and low toxicity, among others, leading to an enhanced selectivity for monovalent anions [27,28].

Moreover, several studies have reported an improved rejection of multivalent ions such as sulfate (usually expressed in RED studies as the permselectivity between Cl^−^ and SO_4_^2−^) by different modified AEMs [29]. For instance, a Fujifilm AEM Type I was modified via LbL deposition, reaching an improved permselectivity between Cl^−^ and SO_4_^2−^ from 0.81 to 47.04 after modification [20]. On the other hand, a Neosepta AMX AEM modified with polydopamine (PDA) by dip coating was reported to be capable of decreasing the permselectivity between SO_4_^2−^ and Cl^−^ from 1.2 to 0.22 [30], thus denoting that the transport of SO_4_^2−^ across AEMs can be controlled by their surface modification.

The scientific community is, however, continuously seeking to investigate innovative approaches in an attempt to design tailor-made modified AEMs respecting greener and more sustainable preparation/utilization ways. Thus, the development of cheaper, environmentally friendly, non-toxic, stable, hydrophilic, and durable materials for AEMs functionalization still represents a real challenge to be solved. In this context, as the LbL method represents a subsequent addition of negatively and positively charged layers on a membrane surface, more toxic polymers/substances are involved in the formation of positively charged layers, which leads to a certain loss of sustainability related aspects.

Therefore, we propose here the monolayer modification of AEMs with poly(acrylic) acid (PAA), which is a cheap, eco-friendly, and non-hazardous substance. In addition, due to the negative charge of most of PAA chains in aqueous solutions at neutral pH, this polyelectrolyte can be easily used to create a negative hydrophilic layer on AEMs, which may exhibit antifouling features and improved monovalent anion permselectivity. PAA has been previously used as a model foulant [31] as well as for improving membrane hydrophilicity [32,33]. However, to the best of our knowledge, our study is the first attempt reported in literature to functionalize the surface of AEMs with PAA for RED related purposes. In this respect, a comprehensive characterization of membrane behavior is essential to move forward on the development of novel modified membranes for blue energy harvesting [34].

Consequently, this work focuses on the comprehensive characterization of PAA-modified heterogeneous Ralex-AEMs, which are strongly basic AEMs with quaternized ammonium functional groups [35] with a significantly lower cost compared to that of homogenous AEMs. As known, one of the main current limitations for commercialization of the RED process is the relatively high costs of the IEMs, thus cheaper materials have to be considered. The monovalent anion permselectivity and fouling of the prepared membranes in the presence of humic acid (HA) through mass transport experiments is also assessed. The behavior of the modified membranes as a function of PAA concentration is compared with the performance of unmodified commercial AEMs, thus providing new insights and knowledge for the continuous research, design, and development of functionalized AEMs for an improved RED process operation.

## 2. Materials and Methods

### 2.1. Membrane Preparation

Commercial polyester-based heterogeneous Ralex-AEMs (MEGA, Stráž pod Ralskem, Czech Republic) were modified by putting them in contact with PAA-based solutions for 24 h, where both conductivity and pH of the PAA-modifier solution were monitored with time during modification. Trizma^®^ (Sigma-Aldrich, St. Louis, MO, USA) buffer aqueous solutions (0.1 M) including different PAA concentrations (from 1 to 5 g/L) were used to create a negatively charged monolayer onto the membrane surface. After functionalization, the solution was replaced by fresh 0.1 M Trizma^®^ solution to carry out membrane cleaning for 24 h. The resulting modified membranes were kept in water (total immersion in deionized water) before characterization and/or use. Table 1 shows the classification, modification conditions, and nomenclature of the membranes under investigation.

### 2.2. Membrane Surface Characterization

Surface hydrophilicity was evaluated by using a goniometer (CAM 100, KSV Instruments Ltd., Helsinki, Finland) together with a software for drop shape analysis. In the experiment, a droplet of deionized water was provided by means of a syringe onto membrane surface (membranes dried at 35 °C for 24 h were used), where different (at least four) surface points were taken into consideration to reach an averaged contact angle value in each case, including the standard deviation.

The water uptake (*WU*) of the prepared AEMs was measured by weighing membrane mass at dry (*m_dry_*) and wet (*m_wet_*) conditions, respectively. Firstly, AEMs were dried at 35 °C for 24 h, followed by using a desiccator for 1 day to remove traces of water. Finally, the membranes were totally immersed in deionized water for 24 h to obtain the wet mass of the corresponding AEMs, after removing the excess of water from membrane surface with a tissue paper. The *WU* percentage is then calculated as follows:

(2) $$WU\;\left(\%\right)=\frac{m_{wet\;}-m_{dry}}{m_{dry}}\times 100$$

The amount of fixed charged groups per unit weight (g) of dry polymer in the prepared AEMs, that is, their ion exchange capacity (*IEC*), was firstly measured by adapting the Mohr titration method [36]. Wet AEM samples with known masses were immersed in a 0.4 M NaCl aqueous solution for 24 h. The anion exchange is carried out by replacing the former solution by 0.2 M Na_2_SO_4_ aqueous solution, which was kept in contact with the AEMs for 3 h, thus replacing Cl^−^ by SO_4_^2−^. The resulting solution containing the released Cl^−^ was finally titrated using a volumetric 0.1 M AgNO_3_ aqueous solution (potassium chromate was utilized as indicator) to calculate *IEC* values in mmol per mass (g) of dry membrane as a function of the type of AEM under study as follows:

(3) $$IEC\;\left(mmol/g_{dry}\right)=\frac{V_{AgNO_{3\;}}\times N_{AgNO_{3\;}}\;}{m_{dry}}$$

A spectrophotometric IEC determination method, proposed in [37], was also performed for the sake of comparison. The membrane samples were immersed in 1 M KNO_3_ aqueous solution for 24 h, followed by immersion in 0.1 M NaCl for 12 h in order to exchange Cl^−^ for NO_3_^−^, which is released to the solution. The concentration of NO_3_^−^ was measured using a UV-visible spectrophotometer (Thermo Scientific Evolution 201, ThermoFisher Scientific, Waltham, MA, USA) that operates at a wavelength of 300 nm, thus determining the IEC taking into consideration the measured NO_3_^−^ (mmol) and the averaged mass of the dry membranes.

The membrane fixed charge density (*CD_fix_*), which represents the concentration of fixed charge groups per unit volume of water in the membrane under study, was estimated by the relation between the *IEC* and the *WU* as follows: *CD_fix_* = *IEC/WU*.

The swelling effect was also evaluated by measuring the thickness (Elcometer 124 Thickness gauge, Elcometer Instruments, Manchester, UK) and diameter of the prepared membranes in both wet (AEMs in contact with deionized water for 24 h) and dry (AEMs at 35 °C 24 h, followed by using a desiccator for 1 day) conditions.

The analysis of functional groups at the membrane surface was carried out by Fourier attenuated atomic force microscopy (*ATR-FTIR*) technique using a Spectrum Two FT-IR Spectrometer (PerkinElmer^®^, Waltham, MA, USA). Due to operational requirements, the analyzed AEMs were dried (in an oven at 35 °C for 24 h) and kept in a desiccator for 1 day before use. The response of unmodified heterogeneous Ralex-AEM was also measured as a reference. At least three points (different positions) of a membrane surface were analyzed to obtain reproducible spectra in each case.

### 2.3. Electrochemical Characterization

The prepared AEMs were firstly electrochemically characterized by cyclic voltammetry (*CV*) using a divided two-compartment diffusion cell, where the effect of the type of electrodes and their relative position was studied in order to reach ideal resistor performance. For that purpose, copper (Cu), graphite, and silver (Ag) rods were taken into consideration as electrodes in this study. The feed compartment (containing the counter electrode) was filled with an aqueous solution of 1 g/L NaCl + 0.1 g/L Na_2_SO_4_ (including the effect of the presence of 25 ppm of HA), whereas 30 g/L NaCl (mimicking a seawater salinity) was utilized at the receiver compartment (containing the working electrode) to perform the electrochemical measurements from −0.6 to 0.6 V (a scan rate of 200 mV/s was used). The potential was controlled by using a potentiostat/galvanostat (Ivium Technologies, Eindhoven, Netherlands). Three repeated scans were carried out in all tests to study membrane and diffusion stability. In this context, the obtained electrochemical responses are related to the overall transport of ions crossing the corresponding AEM under study. On the other hand, the current obtained at the maximum applied voltage (0.6 V) was evaluated in four different feed aqueous solutions with the same molar concentration (i.e., 0.017 M + 0.0007 M) such as KCl + Na_2_SO_4_, KCl + K_2_SO_4_, NaCl + Na_2_SO_4_, and LiCl + Na_2_SO_4_, respectively, in order to study the effect of the nature of the co-ion.

**Figure 1:**
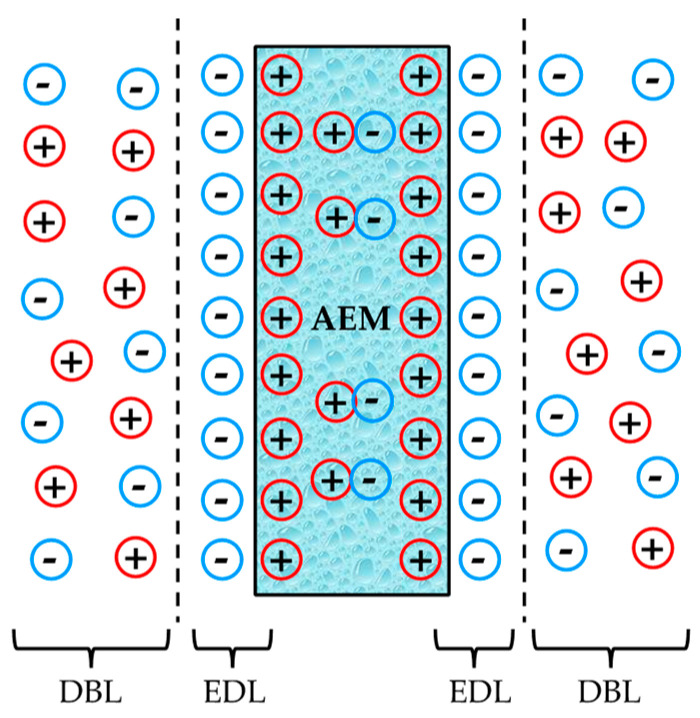
Anion exchange membrane diagram including both electric double-layer (EDL) and diffusion boundary layer (DBL) effects, adapted with permission from [40]. Copyright 2016 Elsevier.

A dedicated, compact, and robust electrochemical flow cell designed by Østedgaard-Munck et al. [38,39] was used to characterize the AEMs through electrochemical impedance spectroscopy (*EIS*) measurements, aiming at studying membrane electro-resistance. The redox flow cell consists of two symmetrical halves separated by the membrane under study. The geometric active area of the cell is 6.25 cm^2^. One stainless steel end plate is placed as a final element at the end of each cell side. The end plates are electrically insulated from gold plate-based current collectors by using a Viton sheet. Graphite blocks act as electrodes, which are intentionally designed with an interdigitated flow pattern based on 12 channels with a dimension of 25 × 25 × 2 mm. Additionally, two Teflon gaskets were located between the electrodes and the membrane to ensure the correct adjustment of the key element of the system. The two feed streams (0.5 M NaCl aqueous solutions) were supplied in co-flow mode to the cell at 20 mL/min by using a peristaltic pump (Masterflex, Cole-Palmer, Chicago, IL, USA). Besides 0.5 M NaCl solutions, 0.5 M KCl and 0.5 M LiCl solutions were also considered to evaluate the effect of changing the co-ion on the membrane electro-resistance. Finally, the cell is specifically tightened at a torque force of 3.0 Nm using a torque wrench, which allows an optimal contact between the electrodes, the membrane, and the different elements in the redox flow cell. Impedance analyses were carried out at room temperature and constant voltage (50 mV) with an amplitude of 0.1 V using the same Ivium potentiostat described for *CV* measurements, with frequencies ranging from 0.5 MHz to 100 Hz. Different EIS measurements at the same conditions were run to reach an averaged membrane electro-resistance, including blank experiments (EIS experiments without membrane). The data was fitted by means of equivalent circuit analysis to determine the effect of the electric double-layer (EDL) in each case, which relates to the structure of charge accumulation and charge separation that occurs at the interface between the membrane and the aqueous solution-based electrolyte, as shown in Figure 1. Due to the high frequency range used in this study, the effect of the diffusion boundary layer (DBL), also represented in Figure 1, should be lower than the one associated with the membrane resistance, even though it has also been assessed for the sake of clarity. Therefore, we focus the *EIS* investigation on (i) membrane electro-resistance (R_M_), and (ii) electric double-layer resistance (R_EDL_).

### 2.4. Mass Transport Experiments

**Figure 2:**
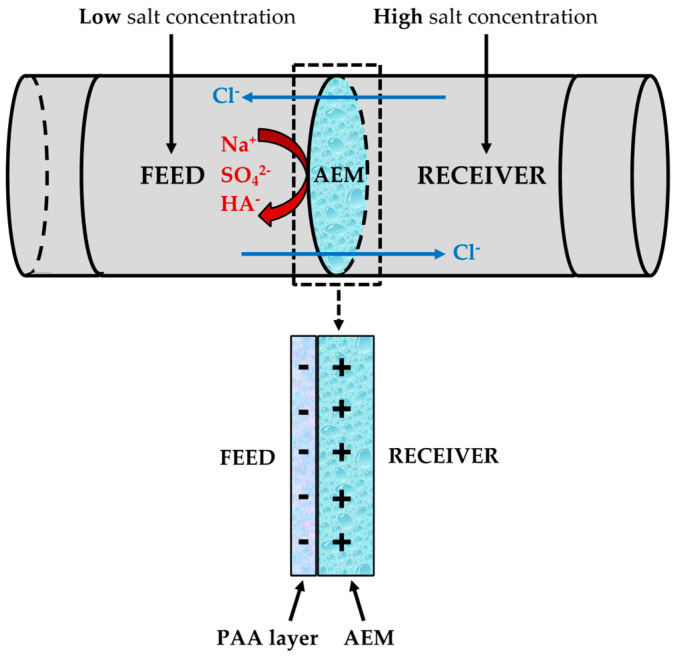
Two-compartment diffusion cell layout.

With the purpose of evaluating the counter-ion permselectivity and fouling behavior of the prepared AEMs, the same two-compartment diffusion cell used for membrane modification and CV analyses was utilized to carry out mass transport studies. Thus, the feed and the receiver compartments were filled with model streams of low (i.e., river water) and high (i.e., seawater) salt concentrations, while 25 ppm of HA (Fluka, Ign. residue: ≈20%) was introduced in the feed solution in several experiments as model organic foulant to evaluate fouling behavior. Figure 2 shows the diffusion cell layout, where it is worth noting that no potential difference is applied to the system to conduct the diffusion experiments. The time evolutions of concentrations of the ions present (Na^+^, SO_4_^2−^ and Cl^−^) in both compartments were followed by taking and analyzing samples for 24 h. Na^+^ and SO_4_^2−^ concentrations were determined using an Inductively Coupled Plasma (*ICP*) Spectroscopy technique (Na and S determination) and further calculations for SO_4_^2−^, whereas Cl^−^ was calculated using charge balance difference. In the presence of HA, the absorbance of feed and receiver solutions was studied by using the UV-visible spectrophotometer mentioned above to demonstrate that HA (absorbance at 280 nm) is not permeating through the membrane from feed to receiver compartment.

## 3. Results and Discussion

### 3.1. Membrane Surface Characterization: Contact Angle, Water Uptake, Ion Exchange Capacity, Fixed Charge Density, Swelling, and Fourier Attenuated Atomic Force Microscopy

**Figure 3:**
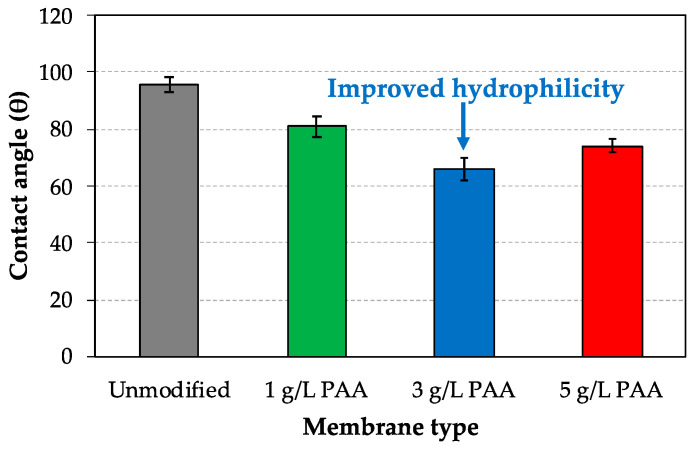
Hydrophilicity analysis: contact angle data.

Surface hydrophilicity was evaluated by using a goniometer integrated with a software for drop shape analysis, where the lower the contact angle of a membrane, the higher its hydrophilicity. Thus, Figure 3 shows contact angle values for different one side PAA modified AEMs and their comparison with the unmodified (commercial) membrane, demonstrating an improved membrane hydrophilicity after modification with 1, 3, and 5 g/L of PAA-based solution respectively. In particular, the deposition of a 1 g/L of PAA-based layer involves an improvement of 15% in hydrophilicity in comparison with the contact angle value achieved for the unmodified heterogeneous AEM, while increasing the PAA concentration up to 3 g/L during modification step results in modified AEMs with higher hydrophilic properties (31% enhanced). However, a further increase in the PAA concentration to 5 g/L did not enhance the hydrophilicity results reached at 3 g/L, which might be possibly due to surface saturation at higher PAA concentrations. It is worth highlighting that different positions on the surface of the dried membranes under investigation were considered to reach averaged contact angle values.

The membrane hydrophilicity improvement for the PAA-modified AEMs was confirmed by water uptake (*WU*) analyses. Table 2 summarizes *WU*, ion exchange capacity (*IEC*) via two different methods, and fixed charge density (*CD_fix_*) results of the prepared membranes as a function of PAA concentration, including the data associated with the unmodified membrane. In this regard, the modified AEMs present higher WU values compared to the unmodified membrane, denoting the higher hydrophilic properties of the membranes, for which an additional negative PAA layer was incorporated onto their surfaces, even though alterations in PAA concentration did not involve significant (taken into account the standard deviation) changes in WU values, thus denoting comparable water absorption properties of the prepared PAA-modified AEMs.

Membrane composition affects *IEC* due to the presence of different fixed functional groups, which can be divided into weak and strong ion-exchangers according to their dissociation constants [37]. Two distinct methods were considered to evaluate the *IEC* of the prepared membranes, as shown in Table 2. The results obtained demonstrate that the determined *IEC* values are dependent on the method applied, which suggests that care should be taken when selecting the most appropriate analytical technique in case of surface modified membranes. With the Mohr titration technique (method 1), *IECs* from 0.3 to 0.95 mmol/g were obtained. As expected, the PAA modified AEMs presented lower *IEC* values, in comparison with the unmodified membrane. As the concentration of PAA during modification step increases, a stronger repulsion effect of the negative PAA layer on SO_4_^2−^ occurs, leading to a reduced anion exchange/replacing between Cl^−^ and SO_4_^2–^ and, respectively, lower *IEC* values determined in this way.

On the other hand, when spectrophotometric measurements (method 2) are conducted, *IEC* values from 1.3 to 1.8 mmol/g were achieved as PAA concentration increases. In this regard, *IEC* values are slightly higher for the modified AEMs compared to those for the unmodified membrane, even though the influence of PAA concentration seems to be negligible at higher concentrations, which involves a similar replacing between Cl^−^ and NO_3_^−^ in the different modified membranes. Therefore, this study highlights the importance of the method adopted to evaluate *IEC* in ion exchange membranes, thus demonstrating that *IEC* depends on the selected ion for replacement/exchange. In this context, Mohr titration and visualization methods usually involve higher errors due to the difficulty to determine the final equivalent point by a naked eye. In this relation, spectrophotometric methods could determine more accurate *IEC* values (similar to those obtained via elemental analysis) according to a study, in which several methods for determining *IEC* of AEMs have been discussed and compared [37]. As a result, we recommend spectrophotometric approaches to determine the *IEC* of surface modified AEMs. Regarding the *CD_fix_* results, this characterization parameter is affected by both *IEC* and *WU*. Since different *IEC* values were observed as a function of the method used, *CD_fix_* results follow the same tendency as the one observed for the *IEC*.

Membrane swelling is another essential parameter that may negatively affect the performance of RED because, for instance, the thickness of the membrane may increase its electrical resistance, leading to reduced power output from RED. Despite the fact that swelling degree is often measured as water uptake in literature [36,41], it is worth mentioning that membrane swelling must also be quantified in terms of membrane dimensional changes. Therefore, in this work the effect of swelling on both membrane thickness and diameter is evaluated. Thus, Table 3 shows not only the study of the mentioned dimensions for the prepared PAA-modified AEMs after and before swelling, that is, under wet and dry conditions, respectively, but also including the behavior of the unmodified commercial Ralex membrane.

The thickness of the modified PAA-AEM is slightly higher at both dry and wet conditions in comparison with the values obtained for unmodified membranes, denoting the effect of the additional negative PAA layer incorporated onto membrane surface. Significant changes can be observed in the thickness of the membranes under investigation after swelling, denoting an increase of the thickness of swelled membranes of around 36–38% compared to their thicknesses at dry conditions, which demonstrates the essential role of the membrane operating conditions for improved RED performance, since higher thicknesses might result in increased membrane electro-resistance, thus reducing the obtainable net power density. On the other hand, focusing on the study of diameter differences, a similar trend is observed, even though the values are increased after swelling by 5% in most of the cases. These dimension changes suggest that either the thickness and diameter of the membranes might be affected by swelling conditions, which may involve alterations in membrane electro-resistance, permselectivity and, therefore, RED process efficiency. Overall, this analysis demonstrates the importance of controlling the dimensions of AEMs with the purpose of optimizing membrane design and operating conditions for RED applications.

**Figure 4:**
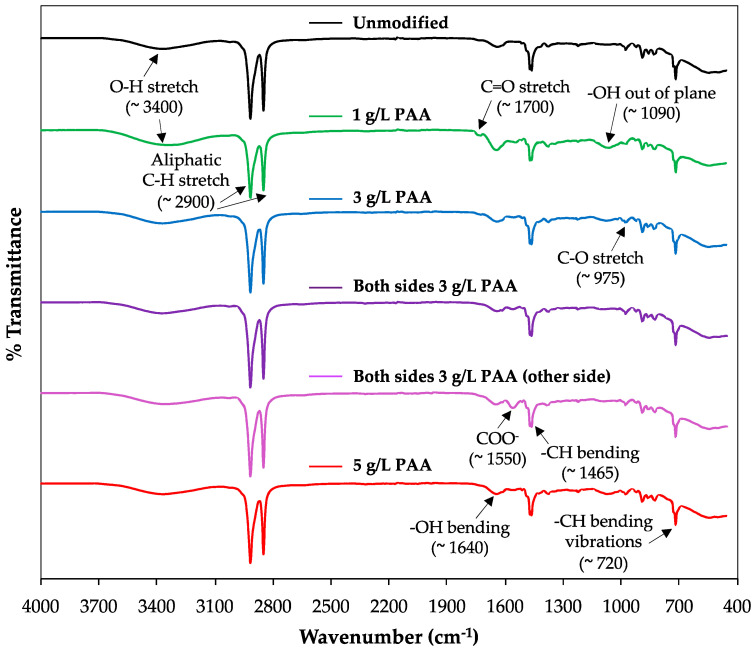
Fourier attenuated atomic force microscopy (*ATR-FTIR*) spectra of unmodified and poly(acrylic) acid (PAA)-modified AEMs.

The analysis of functional groups via *ATR-FTIR* spectra is shown in Figure 4, where both the responses of unmodified and one side/both sides PAA-modified AEMs are presented in an attempt to demonstrate successful membrane modification and stability.

Similar *ATR-FTIR* profiles can be observed for the modified and unmodified membranes, which might be explained partially by taking into account that the amount of the modifying agent at the membrane is not enough to produce significant changes in *FTIR* spectra. This fact suggests the absence of chemical reactions between the PAA and the membrane, thus demonstrating that the attachment is electrostatic. Consequently, since PAA is not covalently bound to the membrane, the amount of PAA before membrane washing with Trizma^®^ solution must be higher and, therefore, *FTIR* signals for the PAA-modified AEMs should be stronger, helping to easily identify the expectable contribution of PAA to the FTIR spectra. However, the washing step is essential because the goal is to remove the loosely attached PAA, avoiding the release of this modifying agent during the process while ensuring membrane stability. Additionally, further difficulties in accessing the presence of PAA are present because both, Ralex membrane and PAA, have similar groups.

Thus, the different bands/peaks observed in the spectra correspond to the polyester fabric (Ralex AEM) and the PAA used for creating the negative monolayer on membrane surface. For instance, the first band that can be observed at ≈3400 cm^−1^ is related to the O–H stretch of the carboxylic group that is present in both polyester and PAA, whereas the next two peaks at around 2900 cm^−1^ are associated with the C–H stretch of the two substances, corresponding to –CH_2_ and –CH_3_ groups. At lower wavenumbers, the C=O stretch can also be identified (≈1700 cm^−1^), which relates to polyester and to the carboxylic group of the PAA. Besides, the sharp bands at around 1640 and 1465 cm^−1^ correspond to PAA and polyester as –OH and –CH bendings, respectively [42,43]. At 1090 cm^−1^, the “shaped like U” peak might be either associated to -OH out of plane (carboxylic group) or O=C–O–C stretching of the main polymer of the membrane [43,44]. In this regard, the band is more visible in the modified membranes compared to the response achieved for unmodified AEMs (black spectrum), which the authors relate to the inclusion of an additional –OH group during surface modification with PAA. On the other hand, the C–O stretch referred to the glycol was also observed in the six spectra at 975 cm^−1^, approximately [44], including the signal of C=C stretching related to the benzene ring of polyester at a similarly wavenumber. Moreover, –CH bending vibrations signal was also identified at 720 cm^−1^ [45]. Since the pH of the solution during modification was higher than 6.4, the dissociation of COOH into COO^−^ and H^+^ is clearly presented in the PAA-modified membranes at ≈1550 cm^−1^ [46], which evidences the presence of PAA on the modified membrane surfaces. This *ATR-FTIR* analysis, therefore, demonstrates the high chemical stability of the polyester-based AEMs after adding a negative PAA monolayer onto their surfaces.

### 3.2. Electrochemical Characterization: Cyclic Voltammetry and Impedance Spectroscopy Measurements

The electrochemical characterization of the prepared AEMs was firstly evaluated through *CV* measurements. Firstly, the one side modified membrane with a PAA concentration of 1 g/L was selected (owing to its higher water uptake properties) in an attempt to evaluate its electrochemical response as a function of (i) type of electrodes, (ii) relative electrodes position in the diffusion cell (feed or receiver compartment), and (iii) HA presence, with the purpose of determining the optimum conditions for RED process operation. In this regard, Cu electrodes were first employed in this study, but due to the problem of Cu oxidation at this potential window (i.e., −0.6–0.6 V) as well as the low current responses achieved, the application of graphite and Ag electrodes was further evaluated. However, the application of graphite electrodes in both compartments resulted in poor electrochemical responses as well, which can be associated with the low conductivity of this material at room temperature, even though the combination of a graphite electrode with a silver rod one resulted in an improved membrane behavior in terms of current-voltage response. Nevertheless, none of the possible combinations between Ag and graphite rods allowed us to reach the ideal resistor performance. This behavior, however, was reached at Ag-Ag electrodes due to the high stability and conductivity of this material in aqueous solutions. Besides, it is important to highlight that this combination is able to reach zero current at zero voltage, approximately, which is essential from an electro-membrane process point of view. Moreover, the presence/absence of HA did not affect the current voltage profile, denoting the negligible impact on the overall transport of ions across the membrane under investigation, although the monovalent permselectivity of the membrane can be reduced (negatively affected) in the presence of HA. Therefore, this Ag-Ag combination was selected to be used in further *CV* characterization analyses.

**Figure 5:**
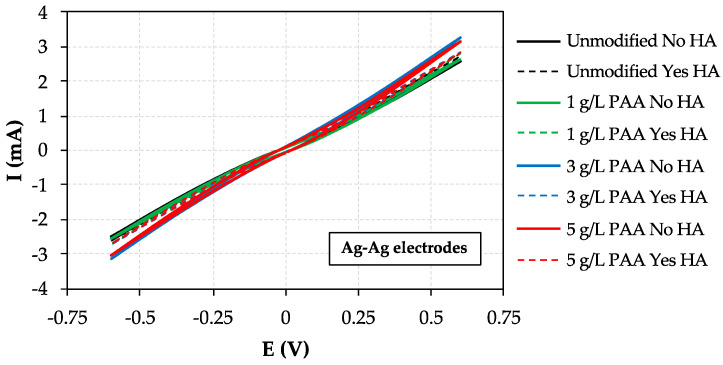
Cyclic voltammetry analyses: effect of PAA concentration in AEM modification at Ag electrodes.

Thus, the effect of PAA concentration during modification step as well as the comparison between the different one side modified AEMs and the unmodified one in terms of current-voltage (I-E) behavior was also investigated in the presence/absence of HA, as presented in Figure 5. No significant changes were observed in terms of current-voltage profiles for all membranes, even though the PAA-modified AEMs presented higher current responses due to their improved hydrophilic properties, as demonstrated by the contact angle and water uptake data obtained, which results in a higher overall transport of ions through the corresponding modified membranes. It is also worth noting that very similar currents were achieved at −0.6 V for the AEMs modified with 3 and 5 g/L of PAA, respectively. The best membrane performance, that is, the one which is able to reach higher currents at −0.6 V, was observed to be the modified AEM with 3 g/L of PAA-based solution (in the absence of HA), denoting that there is an optimum PAA concentration level in terms of overall ions transport and behavior for RED. Moreover, the low overall current-voltage responses achieved in the presence of HA (25 ppm at the feed compartment), especially at high PAA concentrations (i.e., 3 and 5 g/L) may be explained by considering the fact that part of the HA is deposited onto membrane surface, this reducing the overall transport of ionic species through the membrane under investigation. This issue is further evaluated at the end of this section via mass transport experiments. It is also worth noting that linear sweep voltammetry (*LSV*) tests were carried out at the same conditions to confirm that the I-E curves behave the same as the stable cycles reached via *CV* analyses.

Besides, with the purpose of evaluating the effect of the nature of the co-ion present in the feed compartment (simulating river water streams), Table 4 reports the current obtained at the maximum applied voltage (i.e., 0.6 V) for different membranes (i.e., unmodified, one side 3 g/L modified and one side 5 g/L modified AEMs) as a function of the monovalent salt involved in the feed electrolyte composition.

As it can be observed by comparing the currents reached for the different feed compositions due to the increasing hydrated ionic radii in the order K^+^ < Na^+^ < Li^+^, the registered currents decreased in the same order (Table 4). This behavior is clearly shown regardless whether the membranes were modified or not, which could be attributed to the more efficient Donnan exclusion of more hydrated (i.e., with bigger sizes) co-ions from the AEMs.

Furthermore, *EIS* experiments were carried out at a constant voltage of 50 mV and high frequency levels (0.5 MHz to 100 Hz) to focus on the following two key parameters: (i) membrane electro-resistance (R_M_) and (ii) electric double-layer resistance (R_EDL_), both obtained at high and moderate frequencies considering the frequency range of the scope of this study, respectively. Although the diffusion boundary layer resistance (R_DBL_) is often neglected under these operating conditions, its effect has also been evaluated in this comprehensive study for the sake of clarity.

**Figure 6:**
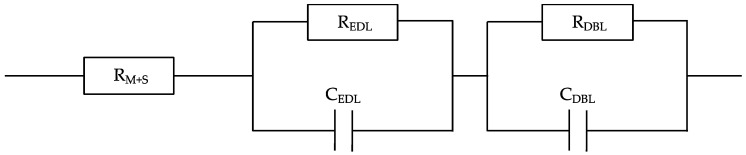
Equivalent circuit diagram showing the combination of membrane, electric double-layer, and diffusion boundary layer resistances.

The equivalent circuit analysis tool was used to fit the results obtained from EIS measurements in order to obtain the three target parameters. In this respect, the equivalent circuit which better fits the data obtained with high accuracy for the unmodified and the modified membranes is associated with the series and parallel combination of a resistor (R) and two capacitors (C), respectively, as shown in Figure 6. At higher frequencies (i.e., ≥10 kHz), the real impedance measured at zero phase shift represents R_M_.

**Figure 7:**
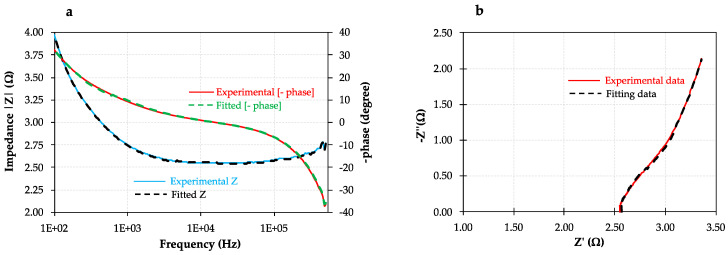
Electrochemical impedance spectroscopy (*EIS*) study of the unmodified membrane including experimental (continuous line) and fitting data (dotted lined): (**a**) Bode plot; (**b**) Nyquist plot.

In this context, R_M_ was firstly experimentally calculated by subtracting the electrical resistance of the blank experiment (solution flowing without membrane in the redox flow cell) to the combined resistance between the membrane and the solution (R_M+S_) at zero phase shift. Each experimentally measured R_M_ value was compared and validated with the value obtained by using the equivalent circuit model provided by the Ivium apparatus incorporated software. The evolution of the experimentally measured impedance and phase shift (φ) of the unmodified AEM as a function of frequency range is represented by the Bode plot (Figure 7a) as an example, where three replicates (same membrane) at the same conditions were run to obtain the key parameters with accuracy (i.e., averaged R_M_, R_EDL_, and R_BDL_), including the fitting results for the sake of comparison. Taking into account the experimental data and the equivalent circuit analysis at zero phase shift, the electrical resistance of the unmodified membrane (R_M_), considering the geometric active area of the redox flow cell was 5.01 ± 0.52 Ω·cm^2^ (at 1.7 × 10^4^ Hz). Subsequently, both R_EDL_ and R_DBL_ were obtained using the equivalent circuit tool provided by the Ivium software of the potentiostat. In this regard, the R_EDL_ was found to be significantly lower than membrane resistance, reaching an average value of 1.83 ± 0.23 Ω·cm^2^ for the frequency range from 500 to 1.7 × 10^4^ Hz, which denotes the fact that the restriction of ions transfer is higher in the membrane. Finally, the effect of the DBL (observed in the frequency range of 100–500 Hz) was also quantified in terms of resistance (R_DBL_), achieving an averaged value of 0.74 ± 0.15 Ω·cm^2^, which represents a considerably lower resistance value compared either with R_M_ and R_EDL_, as previously expected due to the frequency range considered.

On the other hand, Figure 7b shows through the Nyquist plot both real (Z′) and imaginary (Z″) impedances, which were measured for the unmodified membrane.

In this work, a part of the typical well-defined semicircle in Nyquist plots is appearing due to the frequency range from 0.5 MHz to 100 Hz considered in this study in order to avoid possible salt accumulation in the graphite blocks of the electrochemical cell at low frequencies (personal communication to the authors). However, the mentioned accuracy of the equivalent circuit considered for the determination of the target parameters was high enough as demonstrated by the X^2^ error function (0.002 approximately).

The averaged membrane electro-resistances of the different tested membranes are shown in Table 5, including both averaged EDL and DBL effects. The small increase in R_M_ when introducing PAA onto the membrane surface might be associated with the higher thicknesses of the PAA-modified AEMs. Besides, R_EDL_ values for the PAA-modified AEMs are closer to the parameter achieved for the unmodified AEM (around 1.8 Ω·cm^2^). Therefore, these small differences in both R_M_ and R_EDL_ are insignificant, thus indicating that the addition of PAA monolayers with different modifying concentrations onto heterogeneous AEM surfaces is not compromising neither the electrical conductivity nor the ohmic/non-ohmic resistances of the different prepared PAA-AEMs under investigation, which is crucial for improving the obtainable net power density from RED. On the other hand, although both R_M_ and R_EDL_ are the dominant resistances of the system, increased R_DBL_ values of up to 0.9 Ω·cm^2^ were expectedly reached at lower frequencies (100–500 Hz) for all membranes. As well known, in RED applications this effect can be reduced by either increasing the flow rate or inducing turbulence [47].

It is also worth noting that one of the prepared membranes (i.e., one side 3 g/L PAA) was selected to evaluate the reproducibility of the modification procedure proposed, which represents an important aspect to be considered for the practical application of the developed modified AEMs. In this context, three different membrane samples were modified (independently) under the same conditions with 3 g/L PAA-based solutions. The three modified membranes were fully characterized (physicochemically and electrochemically), and the standard deviations of the different results are considered in the results referred to the membrane (one side 3 g/L PAA) in the whole manuscript, highlighting the high reproducibility (small standard deviation) of the results obtained for this membrane.

Thus, the results obtained suggest that the PAA is distributed uniformly on the membrane surface, which may lead to a reduced disorderliness and surface heterogeneity, as well as decreased charge transfers. As a result, the capacitance of the electric double-layer and the diffusion boundary layer (C_EDL_ and C_DBL_, respectively) can be obtained from the selected equivalent circuit model with high accuracy according to the low values achieved for the X^2^ error function.

The unmodified membrane and the one side 3 g/L PAA-modified AEM were selected in this study to evaluate the effect of the co-ion through EIS analyses. Table 6 summarizes the *EIS* data obtained as a function of the used aqueous salt solution.

As expected, the membrane electro-resistances decrease following the order LiCl > NaCl > KCl, corresponding to the increasing ionic mobility (i.e., decreasing ionic hydrated radii) in the order Li^+^ < Na^+^ < K^+^. The most relevant data in terms of RED are those for NaCl because of its abundance in seawater.

Finally, in order to compare the membrane resistance results of the present study with the performance of different modified commercial AEMs in terms of this key parameter, Table 7 shows the membrane resistance values (before and after modification) reported in several studies, focusing not only on immersion modification methods (similar strategies to the one proposed in this work), but also taking into account alternative modification approaches.

The application of heterogeneous membranes generally involves a higher membrane electro-resistance in comparison with homogeneous membranes. In this context, the change in their resistance after modification is quite low (see Table 5). By contrast, although the initial (unmodified) membrane resistance of homogeneous AEMs is lower due to their lower thickness and uniform distribution of fixed functional groups, the change in this parameter after modification is considerably higher; in some cases, even higher than that typical for heterogenous membranes. In any case, it is also worth noting, that AEMs with monovalent selective properties are advantageous for RED due to the negative impact of multivalent ions presence on the obtainable net power density. Therefore, there is a trade-off between membrane monovalent permselectivity and membrane electro-resistance which must be optimized in each particular case.

### 3.3. Mass Transport Experiments: Sulfate Rejection Study

**Figure 8:**
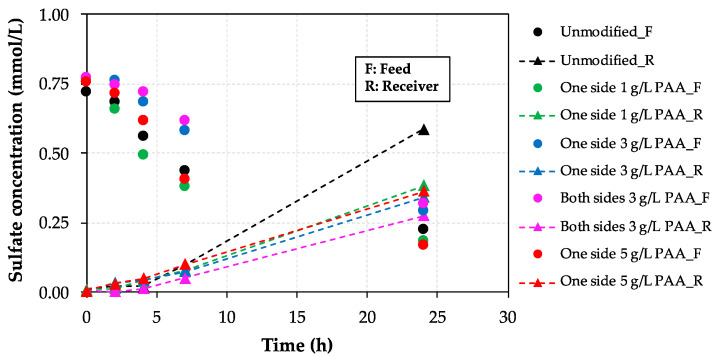
Sulfate evolution with time during mass transport experiments as a function of the AEM used in the absence of humic acid (HA) in the feed compartment.

Mass transport experiments were carried out to evaluate the behavior of the different negatively charged monolayers incorporated onto unmodified AEM surfaces in terms of sulfate rejection, leading to an improved monovalent permselectivity, which would affect favorably the RED process performance. Figure 8 reports the evolution of sulfate concentration with time in both compartments (feed, *F*, and receiver, *R*) as a function of the investigated AEM, including unmodified and modified PAA-AEMs in the absence of HA.

Since at the beginning of the experiment sulfate is only present in the feed (low salt concentration) compartment, the analysis is focused on the evolution of sulfate concentration in the receiver compartment (high salt concentration) for 24 h (dotted lines) in an attempt to follow the sulfate transport through the membrane, thus focusing on sulfate rejection. As can be seen, sulfate rejection is improved as the concentration of PAA increases, although the modified AEM with the maximum PAA concentration (i.e., 5 g/L) did not show the best behavior, which can be associated with hydrophilicity losses as shown in contact angle analysis (see Figure 3). The highest sulfate rejection value is achieved when both sides of the membrane are modified with 3 g/L PAA-based solution (pink dotted line). Thus, the rejection of sulfate is 36%, 42%, 39%, and 54% enhanced for the one side 1 g/L, 3 g/L, 5 g/L, and both sides 3 g/L PAA-modified AEMs, respectively, in comparison with the reached value for the commercial unmodified Ralex-AEM, clearly demonstrating the positive effect of modifying heterogeneous AEMs with PAA solutions to improve the monovalent permselectivity of these membranes for RED applications.

**Figure 9:**
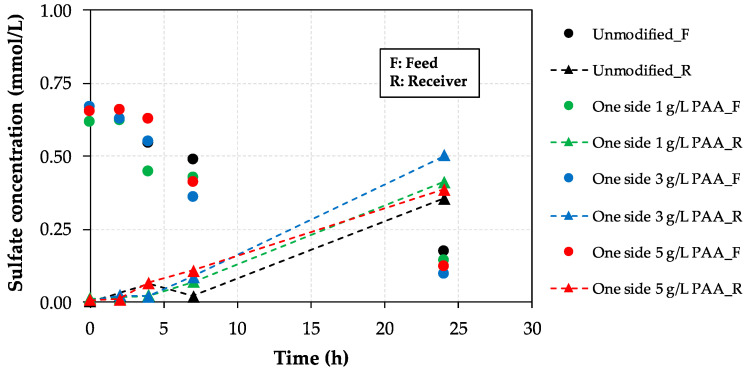
HA studies: sulfate evolution with time during mass transport experiments as a function of the one side modified AEM used.

The presence of HA in the feed compartment during mass transport tests was also investigated, as shown in Figure 9. The presence of 25 ppm of HA in the feed solution involves a negative effect on sulfate rejection owing to fouling phenomena. Surprisingly, the behavior of commercial Ralex AEMs was improved in the presence of HA comparing the concentration of sulfate after 24 h of experiment with the value observed in its absence for the same membrane, denoting behavior changes when a model organic foulant is brought into play. Nonetheless, the performance of the PAA-modified AEMs is worse than that achieved for the unmodified membrane, which demonstrates the high negative effect of fouling phenomena on sulfate rejection and, therefore, on membrane permselectivity. As a result, the concentration of HA in both compartments was monitored with time by measuring the absorbance of each sample at a wavelength of 280 nm. In this respect, it was demonstrated that HA is not crossing the corresponding AEM from the feed compartment to the adjacent one (absorbance very close to zero) in any of the tests examined, which evidences the fact that HA was not present in that compartment. The observed small decrease of HA concentration in the feed solution with time is therefore associated with HA attachment on membrane surface. This phenomenon was confirmed by observing a slight change in the color of the membrane surface (darker orange-like color) that was in contact with the feed solution containing 25 ppm of HA. In short, this study denotes that the behavior of the PAA-modified AEMs is clearly negatively affected by the presence of organic foulants, which would reduce the efficiency of the RED process performance. In this context, the application and study of real natural streams of different salinity, which contains different multivalent ions (i.e., Ca^2+^ and Mg^2+^) as well as different foulants, is essential for further understanding of membrane behavior in order to develop and implement the RED technology at industrial scale.

Moreover, in order to further support the feasibility of the PAA-based modification procedure, the achieved sulfate fluxes (mmol/(m^2^·h)) were estimated through the sulfate concentration time profiles in the receiver compartment for the one side modified AEMs. The concentration differences between 7 and 24 h (when the evolution of the sulfate concentration in time is linear (Figure 8)) were considered for the respective sulfate fluxes calculations and the data obtained are presented in Table 8.

The flux of sulfate was nearly halved for the 3 and 5 g/L PAA modified membranes compared to that for the unmodified membrane, thus confirming the improved Cl^−^/SO_4_^2−^ permselectivity under these operating conditions. The optimal modifying agent (PAA) concentration was equal to 3 g/L.

The future outlook of this research will cover the design, set-up, and long-term operation of a RED stack in order to evaluate the obtainable net power density by using the proposed modified AEMs under real conditions, i.e., using natural feedwaters, which is essential to move forward towards the large-scale implementation of the RED technology.

## 4. Conclusions

In this work, we investigated the complete characterization of poly(acrylic) acid-modified monovalent-anion-permselective membranes for Reverse Electrodialysis applications, where the effect of poly(acrylic) acid concentration during the membrane modification step (from 1 to 5 g/L) was evaluated through several characterization techniques, including mass transport experiments. The following insights can be derived from the results obtained in this study:

- Improved membrane hydrophilicity properties are shown via contact angle analyses for the poly(acrylic) acid (optimal concentration of 3 g/L) modified membranes in comparison with the behavior of the unmodified one.
- The importance of the method used to evaluate the ion exchange capacity of anion exchange membranes is demonstrated, depending on the nature of the replacing anions.
- The swelling effect was investigated in terms of dimension changes (i.e., thickness and diameter). The thickness of swelled membranes is increased by 27%, whereas the diameter is widened by 5% in most of the cases at the same conditions. This analysis highlights the essential role of both thickness (higher thicknesses might result in increased membrane electro-resistance) and diameter (modifications in membrane area may affect process efficiency) to optimize membrane design for RED applications.
- The analysis of functional groups present on membrane surfaces demonstrates the high chemical stability of the polyester-based anion exchange membranes after adding a negative poly(acrylic) acid monolayer onto their surfaces, suggesting the absence of chemical reactions between the modifying agent and the membrane, thus demonstrating that the attachment is electrostatic.
- The use of silver electrodes in cyclic voltammetry measurements allowed to reach ideal resistor behavior. The modified membranes present higher current-voltage responses due to improved hydrophilic properties, which involves a higher overall transport of anions through the corresponding modified membrane, even though the presence of humic acid as model foulant involved a certain decrease in this overall transport owing to its attachment onto the membrane surface.
- The membrane electro-resistances, double-layer resistances, and diffusion boundary layer resistances of the different modified membranes were in the same order of magnitude compared to the unmodified anion exchange membrane (i.e., 5.0–5.4 Ω·cm^2^, 1.6–2.0 Ω·cm^2^, and 0.5–0.9 Ω·cm^2^, respectively) in 0.5 M NaCl aqueous solutions. The small difference observed in the modified ones might be associated with their higher membrane thicknesses. Therefore, the electrical conductivity of the different prepared modified membranes is not compromised by the addition of a negative monolayer onto their surfaces with uniform characteristics, which might involve a reduction of both surface heterogeneity and disorderliness. The membrane electro-resistances decrease in external electrolytes following the order LiCl > NaCl > KCl, owing to the increasing hydrated radii (decreased ionic mobility) in the order K^+^ < Na^+^ < Li^+^. The membrane electro-resistance results obtained in the present study after membrane modification are comparable to those reported in literature for both modified heterogeneous and homogeneous anion exchange membranes.
- Mass transport tests finally prove that the rejection of sulfate (monovalent permselectivity) is improved in the absence of humic acid as the concentration of poly(acrylic) acid increases up to 3 g/L. In this respect, when both sides of the membrane are modified (3 g/L), sulfate rejection is enhanced by 54% compared to the performance of the unmodified membrane, thus suggesting an improved reverse electrodialysis process performance. Nevertheless, the behavior of the modified samples is clearly negatively affected by the presence of organic foulants such as humic acid. The sulfate flux results show that the optimal modifying agent concentration is equal to 3 g/L of poly(acrylic) acid.

Although this study provides new insights and fundamental knowledge for the continuous development of hydrophilic, environmentally friendly, stable, and durable functionalized anion exchange membranes for an enhanced reverse electrodialysis performance, the following challenges, among others, still need to be addressed to make this electro-membrane process feasible/preferred at industrial scale: (i) anion exchange membrane fouling understanding including fouling mechanisms and collective behavior of foulants under natural saline streams conditions, (ii) development of appropriate pre-treatment and cleaning strategies to increase membrane durability and its re-use, (iii) design of greener and cheaper tailor-made anion exchange membranes and modification procedures.

## Figures and Tables

**Table 1:** Anion-exchange membranes (AEMs) under study: classification and characteristics.

List of Membranes	Modification Type/Modified Sides	PAA Concentration (g/L)	Nomenclature
(1)	Unmodified	-	Unmodified
(2)	Monolayer/one	1	One side 1 g/L PAA
(3)	Monolayer/one	3	One side 3 g/L PAA
(4)	Monolayer/both	3	Both sides 3 g/L PAA
(5)	Monolayer/one	5	One side 5 g/L PAA

**Table 2:** Water uptake (*WU*), ion exchange capacity (*IEC*), and fixed charge density (*CD_fix_*) results of the prepared AEMs.

Membrane Type	*WU* (%)	*IEC*^1^ (mmol/g)	*IEC*^2^ (mmol/g)	*CD_fix_*^1^ (mmol/g)	*CD_fix_*^2^ (mmol/g)
(1) Unmodified	59.0 ± 1.8	0.949 ± 0.18	1.369 ± 0.05	1.605 ± 0.25	2.321 ± 0.08
(2) One side 1 g/L PAA	67.4 ± 6.0	0.681 ± 0.03	1.625 ± 0.05	1.017 ± 0.14	2.413 ± 0.08
(3) One side 3 g/L PAA	62.7 ± 3.8	0.433 ± 0.01	1.751 ± 0.05	0.692 ± 0.05	2.793 ± 0.08
(4) Both sides 3 g/L PAA	61.5 ± 0.9	0.378 ± 0.01	1.751 ± 0.05	0.614 ± 0.02	2.847 ± 0.08
(5) One side 5 g/L PAA	63.3 ± 5.0	0.352 ± 0.02	1.760 ± 0.05	0.560 ± 0.08	2.779 ± 0.08

^1^ Titration method; ^2^ Spectrophotometric method.

**Table 3:** Swelling study results.

Membrane Type	Thickness Wet (μm)	Thickness Dry (μm)	Diameter Wet (mm)	Diameter Dry (mm)
(1) Unmodified	643.3 ± 5.8	472.7 ± 2.3	45.7 ± 0.6	43.3 ± 1.2
(2) One side 1 g/L PAA	664.0 ± 0.0	487.7 ± 4.0	45.0 ± 1.7	44.8 ± 2.0
(3) One side 3 g/L PAA	654.7 ± 4.6	473.3 ± 2.3	47.3 ± 1.2	44.8 ± 0.8
(4) Both sides 3 g/L PAA	650.0 ± 20.4	473.7 ± 6.0	47.7 ± 1.5	45.5 ± 0.5
(5) One side 5 g/L PAA	654.8 ± 21.3	476.0 ± 5.3	47.3 ± 0.6	45.3 ± 0.6

**Table 4:** Currents obtained at the maximum applied voltage of 0.6 V as a function of the feed composition and the type of membrane. Receiver solution: 0.5 M NaCl.

Membrane Type	Feed Solution	Current (mA) at 0.6 V
(1) Unmodified	KCl + K_2_SO_4_	3.5
	KCl + Na_2_SO_4_	3.2
	NaCl + Na_2_SO_4_	2.7
	LiCl + Na_2_SO_4_	2.6
(3) One side 3 g/L PAA	KCl + K_2_SO_4_	3.4
	KCl + Na_2_SO_4_	3.3
	NaCl + Na_2_SO_4_	3.2
	LiCl + Na_2_SO_4_	2.8
(3) One side 5 g/L PAA	KCl + K_2_SO_4_	3.8
	KCl + Na_2_SO_4_	3.4
	NaCl + Na_2_SO_4_	3.2
	LiCl + Na_2_SO_4_	3.0

**Table 5:** AEM electro-resistances, electric double-layer, and diffusion boundary layer effects in 0.5 M NaCl aqueous solutions, measured through equivalent circuit model tool.

Membrane Type	R_M_ (Ω·cm^2^)	R_EDL_ (Ω·cm^2^)	R_DBL_ (Ω·cm^2^)	C_EDL_ (µF)	C_DBL_ (µF)	X^2^ Error Function
(1) Unmodified	5.01 ± 0.52	1.83 ± 0.23	0.74 ± 0.15	115 ± 16	46 ± 10	0.0023
(2) One side 1 g/L PAA	5.14 ± 0.50	1.98 ± 0.08	0.89 ± 0.14	200 ± 19	93 ± 16	0.0028
(3) One side 3 g/L PAA	5.21 ± 0.04	1.56 ± 0.18	0.67 ± 0.08	188 ± 12	67 ± 8	0.0023
(4) Both sides 3 g/L PAA	5.33 ± 0.16	1.92 ± 0.17	0.53 ± 0.01	72 ± 12	19 ± 4	0.0020
(5) One side 5 g/L PAA	5.36 ± 0.18	1.58 ± 0.02	0.68 ± 0.02	140 ± 15	42 ± 5	0.0023

**Table 6:** EIS results via equivalent circuit model tool in 0.5 M NaCl, 0.5 M KCl, and 0.5 M LiCl aqueous solutions.

Membrane Type		R_M_ (Ω·cm^2^)	R_EDL_ (Ω·cm^2^)	R_DBL_ (Ω·cm^2^)	C_EDL_ (µF)	C_DBL_ (µF)	X^2^ Error Function
(1) Unmodified	LiCl	5.35 ± 0.03	2.32 ± 0.44	0.35 ± 0.00	147 ± 4	59 ± 3	0.0013
	NaCl	5.01 ± 0.52	1.83 ± 0.23	0.74 ± 0.15	115 ± 16	46 ± 10	0.0023
	KCl	4.88 ± 0.02	2.17 ± 0.57	0.30 ± 0.00	166 ± 6	66 ± 5	0.0011
(3) One side 3 g/L PAA	LiCl	5.49 ± 0.02	1.79 ± 0.30	0.49 ± 0.00	145 ± 3	55 ± 2	0.0013
	NaCl	5.21 ± 0.04	1.56 ± 0.18	0.67 ± 0.08	188 ± 12	67 ± 8	0.0023
	KCl	4.87 ± 0.01	1.27 ± 0.11	0.39 ± 0.00	162 ± 2	60 ± 1	0.0011

**Table 7:** Comparison of the membrane resistance values obtained in this study with values reported in literature for other commercial AEMs before and after modification.

AEM Type	Modification Approach	Modifying Agent	R_M_ Before Modification (Ω·cm^2^)	R_M_ After Modification (Ω·cm^2^)	Reference
Heterogeneous Ralex AM-PES (Mega a.s.)	Direct contact/immersion	Poly(acrylic) acid	5.01	5.1–5.4	This study
Heterogeneous AEM (Zhe-jiang Qianqiu Environmental Protection & Water Treatment Co. Ltd.)	LbL deposition	Glutaraldehyde and poly(ethyleneimine)	4.5	4.8	[48]
Neosepta AMX (Astom Corp.)	Dip coating	Polydopamine (PDA)	1.2	2.9	[30]
Neosepta AMX (Astom Corp.)	Immersion	PDA	2.5	5.0	[49,50]
Homogeneous Neosepta ASE (Astom Corp.)	Immersion (co-deposition)	PDA and poly (sodium 4-styrene sulfonate)	3.6	4.5	[51]
Homogeneous JAM-II-07 (Yanrun)	Coating by deposition	Sulfonated reduced graphene oxide nanosheets	3.1	3.7	[52]
Homogeneous Type I (Fujifilm)	Self-adhesion deposition	Sulfonated polydopamine	1.0	6.8	[53]
AEM * (Ionics)	Coating by adsorption	Olygourethane surfactants and disodium salt α, ω-oligooxipropylene-bis(o-urethane-2.4, 2.6 tolueneurylbenzene sulphonic acid)	2.5	5.7	[54]

* No specific membrane name is reported.

**Table 8:** Summary of the sulfate flux results for the one side PAA-modified AEMs as a function of the absence/presence of humic acid in the feed chamber.

Membrane Type	Sulfate Flux (mmol/(m^2^·h))
(1) Unmodified	3.6
(2) One side 1 g/L PAA	2.2
(3) One side 3 g/L PAA	1.9 ± 0.1
(5) One side 5 g/L PAA	1.9
